# Magnetic Resonance Imaging Is an Effective First-Line Noninvasive Tool for Meniscal Tear Detection: A Retrospective Comparative Analysis With Knee Arthroscopy

**DOI:** 10.1016/j.asmr.2024.101065

**Published:** 2024-12-12

**Authors:** Ahmed Mohsen Abbas El-Hagrasy, Aaron Jijimon Theckayil, Mohammad Adeel Khan, Hammad Naqi Khan, Ahsan Javaid Butt

**Affiliations:** a School of Medicine, Royal College of Surgeons in Ireland–Medical University of Bahrain, Busaiteen, Bahrain; b Department of Orthopedics, King Hamad University Hospital, Busaiteen, Bahrain

## Abstract

**Purpose:**

To evaluate and compare the diagnostic accuracy of magnetic resonance imaging (MRI) with intraoperative knee arthroscopic findings for identifying or diagnosing meniscal tears.

**Methods:**

We conducted a retrospective study of patients who underwent MRI and knee arthroscopy showing either medial meniscus (MM) or lateral meniscus (LM) tears at a single university hospital. The preoperative MRI findings of patients were compared with intraoperative arthroscopic findings to determine the presence, location, and morphology of meniscal tears. The results of arthroscopy were considered the definitive diagnosis.

**Results:**

A total of 543 patients were initially identified. Of these, 220 met the study eligibility criteria and were included. The sensitivity, specificity, and accuracy of MRI in relation to arthroscopy were 94.29% (95% confidence interval [CI], 89.05%-97.50%), 78.75% (95% CI, 68.17%-87.11%), and 88.64% (95% CI, 83.68%-92.51%), respectively, for MM tears and 76.74% (95% CI, 66.39%-85.18%), 94.03% (95% CI, 88.58%-97.39%), and 87.27% (95% CI, 82.13%-91.37%), respectively, for LM tears. Complex tears were the most common morphology of tears, and the posterior horn was the most common location.

**Conclusions:**

MRI is an effective first-line noninvasive diagnostic tool for investigating meniscal tears, with overall diagnostic accuracies of 88.64% for MM tears and 87.27% for LM tears. MM tears had the highest incidence, particularly in the posterior horn. MRI showed high sensitivity for MM tears, high specificity for LM tears, and substantial agreement with arthroscopy in diagnosing both MM and LM tears. However, MRI’s specificity for MM tears and sensitivity for LM tears were lower, suggesting that it may not be as reliable in confirming MM tears or ruling out LM tears.

**Level of Evidence:**

Level III, retrospective cohort study.

Meniscal tears are among the most common pathologic conditions of the knee. The prevalence of meniscal tears is approximately 12% to 14%, with an approximate incidence of 61 cases in every 100,000 persons.^1^ Meniscal tears can be broadly classified into traumatic tears versus degenerative tears. Traumatic meniscal tears usually occur in younger populations, who are at risk of traumatic meniscal tears because of participation in forms of contact sports such as football or basketball; however, anyone at any age can be subject to a traumatic meniscal tear.^2^ Degenerative meniscal tears, on the other hand, are typically nontraumatic and are commonly seen in middle-aged or elderly populations owing to naturally occurring aging-related degenerative processes such as knee osteoarthritis.^3^ The medial meniscus (MM) and lateral meniscus (LM) are crescent-shaped fibrocartilaginous structures that function primarily in shock absorption and load transmission through the tibiofemoral joint; therefore, a traumatic twist in the knee—or further damage to an already degenerative knee—may result in a tear to the meniscus.^4^^,^^5^ The rate of meniscal injuries in men is 2 to 4 times higher than that in women.^6^ In the United States, 10% to 20% of orthopaedic surgical procedures involve meniscal surgery, with over 50,000 meniscectomies and 3,000 meniscal repairs performed yearly.^6^

Magnetic resonance imaging (MRI) remains the gold-standard imaging modality and the first-line noninvasive choice for investigating suspected meniscal tears, with arthroscopy being the gold standard for the diagnosis of knee ligament and meniscal injuries.^7^, ^8^, ^9^ In a retrospective study by Magee and Williams,^10^ the sensitivity and specificity of 3.0-T MRI for detecting meniscal tears compared with arthroscopy were 96% and 97%, respectively. In a study by Khalid et al.,^11^ MRI’s sensitivity and specificity in detecting meniscal tears were 94.9% and 85.7%, respectively. A systematic review by Wang et al.^9^ also portrayed high sensitivity and specificity of MRI for meniscal tears, with sensitivity and specificity of 92% and 90%, respectively, for MM tears and 80% and 95%, respectively, for LM tears. Therefore, the widely regarded view of MRI of the knee as a noninvasive alternative to diagnostic arthroscopy is justifiable; however, there is conflicting evidence regarding whether its use should be reserved for complicated cases in which an experienced clinician requires further information to establish a diagnosis or whether it should be part of routine clinical practice for the detection of meniscal tears.^12^, ^13^, ^14^ Previous studies have portrayed that caution should be exercised in the indiscriminate use of MRI scanning for identifying meniscal tears in diagnosing the painful knee given the low specificity seen in cases of concomitant knee pathology.^15^ One study showed that the diagnostic ability of MRI to predict meniscal injuries at acute anterior cruciate ligament (ACL) reconstruction was moderate.^15^ It illustrated its poorest performance in diagnosing LM tears, with MRI failing to detect 97 tears (48.9%) that were found arthroscopically.^15^ Another study concluded that MRI could be a diagnostic tool for meniscal tears but has limited accuracy in classification of the type and location.^16^ MRI is an integral component of the diagnostic workup for patients with suspected meniscal tears. MRI is also used in our health care system as part of the routine diagnostic workup, including preoperative requirements for medicolegal purposes. However, whereas many studies have reported high sensitivity and specificity, others have provided evidence of contexts in which MRI performance may be variable.

Thus, the purpose of this study was to evaluate and compare the diagnostic accuracy of MRI with intraoperative knee arthroscopic findings for identifying or diagnosing meniscal tears. We hypothesized that MRI would be an effective first-line noninvasive choice for investigating meniscal tears in uncomplicated cases with no concomitant pathology.

## Methods

### Study Protocol

This was an observational retrospective cohort chart-review study and was conducted in the department of orthopaedics at a single university hospital. The study used the preoperative MRI radiology reports generated by 3 musculoskeletal radiologists and the intraoperative findings of arthroscopies performed by 5 different orthopaedic surgeons for patients with suspected meniscal tears to determine the accuracy of MRI for diagnosing the presence, type, and location of meniscal tears. The results of arthroscopy were considered the definitive diagnosis; therefore, the results of the MRI reports were assessed accordingly. Ethical approval was received from the overseeing research ethics committee. Additionally, the Standards for Reporting Diagnostic accuracy studies (STARD) guidelines and the CHecklist for statistical Assessment of Medical Papers (CHAMP) were used when writing the report.^17^^,^^18^

This study aimed to capture the cohort of patients from the electronic medical records in the Healthcare Operating Environment (HOPE) database. Patients who underwent knee arthroscopies were initially identified from the HOPE database and were subsequently included if they presented to the accident and emergency trauma clinic or the outpatient orthopaedic clinic with a suspected diagnosis of meniscal tear. These patients were considered eligible for inclusion in the study given that MRI scans were conducted before arthroscopy. The main inclusion criteria for the study included patients whose principal reason for MRI investigation was suspicion of a meniscal tear, patients with a knee injury who underwent arthroscopy, and patients in whom MRI scans were conducted prior to arthroscopic investigation. The exclusion criteria were patients with a history of arthroscopy or knee surgery; patients with a history of intervention before MRI was conducted; patients with concomitant pathology of the knee such as inflammatory conditions (e.g. rheumatoid arthritis, septic arthritis, synovitis, bursitis, or gout) or other ligament injuries (anterior or posterior cruciate ligament); or cases in which there was an inability to classify meniscal tears because of degenerative arthritis radiologically. Additionally, patients were excluded if they had incomplete relevant data from reports, such as age, sex, MRI reports, and arthroscopic reports. Patients were also excluded if the period between MRI and arthroscopy was more than 6 months (180 days).

### Sample Size and Sampling Technique

For sampling of participants, a selective purposive sampling technique was used to sample a total of 220 cases after meeting the inclusion and exclusion criteria set for this study.^19^ The surgical procedure list in the HOPE database was searched for patients undergoing knee arthroscopy or knee debridement for a suspected meniscal tear. Patients included in the database were screened from January 2017 to October 28, 2023, up until the university hospital had transitioned from the HOPE database to a different database, in which radiology reports became unavailable. The total number of patients undergoing knee arthroscopies for meniscal tear injuries was 543 patients. However, only 220 who met the inclusion and exclusion criteria were included in the study.

### Data Collection

After sample selection, relevant data such as age, sex, presenting complaint, medical and surgical history, physical examination findings, MRI scans of patients, and date of knee arthroscopy procedure, as well as arthroscopic findings of the sampled patients, were systematically collected. Available data regarding the presence or absence, morphology, grade, and location of meniscal tears based on both the MRI and arthroscopic findings were collected.

### MRI and Arthroscopy Analysis

All patients had undergone multiplanar, multisequence 1.5-T MRI of the involved knee without intravenous contrast. Images were examined in 3 standard planes: sagittal, axial, and coronal. T2-weighted image (T2WI) and proton density (PD)–fat suppressed (FS) sequences were obtained in the axial plane; T1-weighted image, T2WI, T2WI-FS, and PD-FS sequences were obtained in the sagittal plane; and T1-weighted image PD-FS images were obtained in the coronal plane. MRI images were then studied for the presence or absence, morphology, grade, and location of meniscal tears. Similarly, arthroscopic findings were assessed for the presence or absence of meniscal tears, as well as their morphology, grade, and location. All morphologies, grades, and locations detected on either MRI or arthroscopy were included in the analysis.

### Interpretation of Meniscal Tear Grades on MRI

Various interpretations for the grading of meniscal tears detected on MRI exist; in this study, the grades were defined as follows: grade 0, normal intact meniscus; grade I, intrasubstance globular-appearing signal or small focal area of hyperintensity not extending to the articular surface; grade II, linear increased signal patterns or areas of hyperintensity not extending to the articular surface; and grade III, abnormal signal or hyperintensity intersecting at least 1 articular surface (superior and/or inferior articular surface) of the meniscus, which is considered a definite meniscal tear that is arthroscopically confirmable.^20^^,^^21^ In this study, to allow for an accurate evaluation of the diagnostic accuracy of MRI, grade I and grade II intrasubstance meniscal lesions, along with no tears, were considered negative findings on MRI, whereas only grade III meniscal tears were considered positive findings on MRI. Subsequently, a finding of no tears on arthroscopy was considered a negative result on arthroscopy, whereas detected tears were considered a positive result on arthroscopy.

### Statistical Analysis

All collected data were anonymized, and SPSS software (IBM) was used to calculate the relevant diagnostic values. Categorical variables were expressed in the form of proportions (percentages), whereas continuous data were expressed in terms of means ± standard deviations. The diagnostic accuracy of MRI relative to arthroscopy was analyzed using the sensitivity, specificity, positive predictive value (PPV), negative predictive value (NPV), accuracy, and likelihood ratio (LR). Finally, the κ ratio was calculated to assess the agreement between MRI and arthroscopy. *P* < .05 was considered significant.

## Results

**Fig 1:**
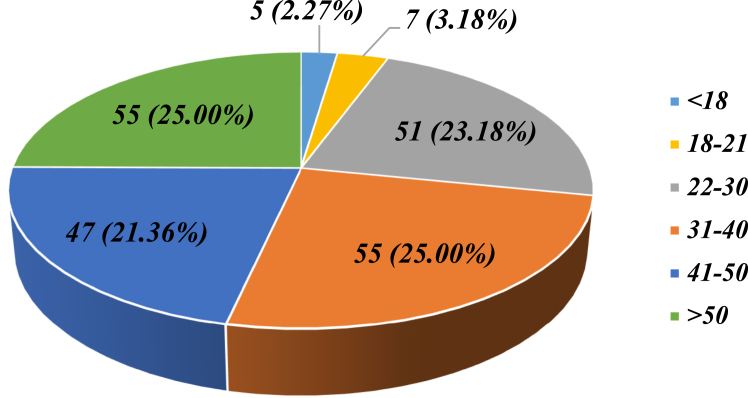
Frequency distribution of cases according to age group in years.

Most of the cases in this study were male patients, with 166 male patients (75.5%) compared with 54 female patients (24.5%), with an approximate 3:1 male-to-female ratio. The age distribution of cases can be seen in Figure 1; most of the patients belonged to the groups aged 31 to 40 years and older than 50 years, each constituting 25.0% of patients.

**Fig 2:**
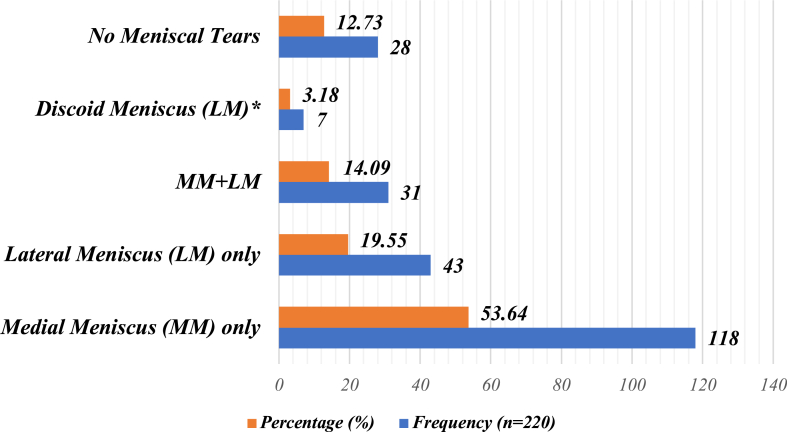
Case-wise distribution of meniscal tears on magnetic resonance imaging. Discoid meniscal tears (asterisk) are considered lateral meniscus (LM) tears and, thus, were excluded from the total frequency and total percentage of meniscal tears; however, they were included to portray the percentage of discoid tears detected. (MM, medial meniscus.)

The incidence of individual MM tears on MRI was 149 cases, present in 67.7% of the 220 total cases. LM tears were present on MRI in 74 cases, comprising 33.6% of the sample, whereas no meniscal tears were visualized on MRI in 28 cases, or 12.7% of the total sample. The case-wise distribution of meniscal tears on MRI can be seen in Figure 2. Most cases (53.6%) had an MM tear only, with the second most common finding being an LM tear alone (19.6%).

### MM Tear

In our study, MM tear was the most common tear observed. Of the 26 cases in which the grade was reported, 9 (34.62%) had grade III tears, 13 (50.00%) had grade II tears, and 4 (15.38%) had grade I tears. The location of MM tears was reported in 145 cases, among which posterior horn tears were present almost exclusively, being reported in 142 cases (97.93%); peripheral tears were reported in 2 cases (1.38%), whereas a tear of the anterior horn was reported in only 1 case (0.69%). The morphology (shape/pattern) of MM tears was reported in 77 cases, with horizontal tears present in 31 (40.26%), followed by complex tears in 26 (33.77%), radial tears in 6 (7.79%), vertical tears in 5 (6.49%), bucket-handle tears in 5 (6.49%), and oblique tears in 4 (5.19%).

The MRI correlation with respect to arthroscopy for MM tears can be seen in Table 1. In our study, 149 cases of MM tear were detected on MRI. Of the 149 cases detected, 132 were confirmed on arthroscopy and thus were true-positive cases whereas 17 were not found arthroscopically and thus were false-positive cases. The remaining 71 cases, in which no MM tears were detected on MRI, were confirmed arthroscopically in 63 cases as true-negative cases, whereas 8 cases were found to be false-negative cases.

**Table 1:** MRI Correlation With Arthroscopy for Medial Meniscal Tears

MRI	Arthroscopy		Total
	Positive	Negative	
Positive	132	17	149
Negative	8	63	71
Total	140	80	220

MRI, magnetic resonance imaging.

Table 2 portrays the statistical correlation between MRI and arthroscopy for MM tears based on the reported diagnostic values. The κ coefficient indicated a substantial or good strength of agreement between MRI and arthroscopy for diagnosing MM tear injuries.

**Table 2:** Statistical Correlation Between MRI and Arthroscopy for Medial Meniscal Tears

Variable	Value	95% CI
Sensitivity, %	94.29	89.05-97.50
Specificity, %	78.75	68.17-87.11
PPV, %	88.59	83.56-92.23
NPV, %	88.73	79.92-93.97
Positive LR	4.45	2.90-6.78
Negative LR	0.072	0.037-0.14
Accuracy, %	88.64	83.68-92.51
κ Coefficient∗	0.748	0.656-0.841

CI, confidence interval; LR, likelihood ratio; MRI, magnetic resonance imaging; NPV, negative predictive value; PPV, positive predictive value.

∗ Approximate significance: *P* < .001 for κ coefficient and 95% CI.

### LM Tear

In our study, LM tears were present in almost one-third of the cases. Of the 19 cases in which the grade was reported, 6 (31.58%) had grade III tears and 13 (68.42%) had grade II tears; grade I tears were not reported. The location of LM tears was reported in 60 cases, among which posterior horn tears were present in 47 cases (78.33%); tears of the anterior horn were reported in 12 cases (20.00%), whereas a peripheral tear was reported in only 1 case (1.67%). The morphology (shape/pattern) of LM tears was reported in 42 cases, with complex tears present in 15 (35.71%), followed by horizontal tears in 8 (19.05%), radial tears in 7 (16.67%), bucket-handle tears in 6 (14.23%), vertical tears in 5 (11.90%), and an oblique tear in 1 (2.38%).

The MRI correlation with respect to arthroscopy for LM tears can be seen in Table 3. In our study, 74 cases of LM tear were detected on MRI. Of the 74 detected cases, 66 were confirmed on arthroscopy and thus were true-positive cases whereas 8 were not found arthroscopically and thus were false-positive cases. The remaining 146 cases, in which no LM tears were detected on MRI, were confirmed arthroscopically in 126 cases as true-negative cases, whereas 20 cases were found to be false-negative cases.

**Table 3:** MRI Correlation With Arthroscopy for Lateral Meniscal Tears

MRI	Arthroscopy		Total
	Positive	Negative	
Positive	66	8	74
Negative	20	126	146
Total	86	134	220

MRI, magnetic resonance imaging.

Table 4 portrays the statistical correlation between MRI and arthroscopy for LM tears based on the reported diagnostic values. The κ coefficient indicates a substantial or good strength of agreement between MRI and arthroscopy for diagnosing LM tear injuries.

**Table 4:** Statistical Correlation Between MRI and Arthroscopy for Lateral Meniscal Tears

Variable	Value	95% CI
Sensitivity, %	76.74	66.39-85.18
Specificity, %	94.03	88.58-97.39
PPV, %	89.19	80.66-94.23
NPV, %	86.30	81.07-90.26
Positive LR	12.85	6.50-25.42
Negative LR	0.248	0.168-0.364
Accuracy, %	87.27	82.13-91.37
κ Coefficient∗	0.726	0.632-0.820

CI, confidence interval; LR, likelihood ratio; MRI, magnetic resonance imaging; NPV, negative predictive value; PPV, positive predictive value.

∗ Approximate significance: *P* < .001 for κ coefficient and 95% CI.

## Discussion

The most important finding of this study is that MRI is an effective first-line noninvasive diagnostic tool for detecting meniscal tears, revealing an overall accuracy of 88.64% and 87.27% for MM and LM tears, respectively. The most commonly impacted age groups were the group aged 31 to 40 years (25.00%) and the group older than 50 years (25.00%). Male patients (75.5%) were affected approximately 3 times more than female patients (24.5%). Consequently, our results exemplify that male sex remains a non-modifiable factor associated with meniscal tears.^22^ Additionally, the most common presenting meniscal injury was MM tear alone (53.64%), followed by LM tear alone (19.55%). In both the MM and LM, the most frequent location of tears was at the posterior horn.

In this study, MM tears had the highest incidence on MRI, with 149 cases (67.7%), being more than twice as common as LM tears, with 74 cases (33.6%). A similar incidence was seen in a study conducted by Antinolfi et al.,^23^ in which there were 49 cases of suspected MM tear and 31 cases of suspected LM tear. This is likely because of the relatively decreased mobility of the MM due to its connection to the medial collateral ligament and its firm attachment to the tibia, especially at the posterior horn.^24^, ^25^, ^26^ The sensitivity, specificity, PPV, and NPV of MM tears on MRI relative to arthroscopy were 94.29%, 78.75%, 88.59%, and 88.73%, respectively, in our study. The specificity and NPV reported in a study conducted by Kim et al.^16^ were almost identical to those in our study, whereas the sensitivity and PPV were found to be higher in our study. The sensitivity was found to be in accordance with that in a study by Khandelwal et al.,^26^ where the sensitivity was higher than the specificity; however, our specificity, PPV, and NPV were found to be lower. Additionally, our sensitivity, PPV, and NPV were found to be higher than those in studies by Bari et al.^27^ and Crawford et al.,^28^ whereas our specificity was found to be lower. The positive LR, negative LR, accuracy, and κ coefficient were 4.45, 0.072, 88.64%, and 0.748, respectively. Our study showed a lower positive LR, accuracy, and κ coefficient than that of Khandelwal et al. but portrayed a similar negative LR.^26^ Moreover, our study showed a higher κ coefficient, indicating greater agreement between MRI and arthroscopy, compared with a study by Nakasa et al.,^29^ in which the κ coefficient was found to be 0.565.

In our study, LM tears were present on MRI in 74 cases (33.6%), which is substantially lower in comparison to previous studies conducted in a similar nature.^16^^,^^23^^,^^26^ The sensitivity, specificity, PPV, and NPV of LM tears on MRI relative to arthroscopy were 76.74%, 94.03%, 89.19%, and 86.30%, respectively, in our study. The specificity of MRI being higher than its sensitivity for LM tears has been similarly portrayed in past studies.^26^^,^^28^ The sensitivity and PPV findings paralleled those of Khandelwal et al.,^26^ and the sensitivity and specificity were nearly identical to those of Crawford et al.^28^ In contrast, our NPV was lower than that reported by Khandelwal et al. In addition, our sensitivity and NPV were very similar to those in the study by Bari et al.,^27^ whereas the specificity and PPV were higher in our study. The positive LR, negative LR, accuracy, and κ coefficient were 12.85, 0.248, 87.27%, and 0.726, respectively. Our study showed a lower positive LR, accuracy, and κ coefficient than that of Khandelwal et al. but portrayed a higher negative LR. Moreover, our study indicated a κ coefficient higher than that in the study by Nakasa et al.,^29^ in which the κ coefficient was found to be 0.333, but lower than the value of 0.838 found in the study by Khandelwal et al.

MRI is an effective first-line noninvasive choice for investigating meniscal tears in uncomplicated cases, with an overall diagnostic accuracy of 88.64% for MM tears and 87.27% for LM tears, adding to its support as a gold-standard imaging modality for meniscal tears. This study shows the importance of MRI in identifying meniscal tears. However, caution is advised when relying on this alone before confirming MM tears and ruling out LM tears owing to MRI’s relatively lower specificity of 78.75% and sensitivity of 76.74% for MM and LM tears, respectively, identified in the study. The high sensitivity of MRI in detecting meniscal injuries, which encompasses its ability to discern subtle details such as intrasubstance tears or degeneration not reaching the surface, may explain why it surpasses arthroscopy’s limitations in viewing only the meniscal surfaces, potentially obviating surgical debridement. This is attributed to MRI’s superior soft-tissue contrast, multiplanar imaging, and high spatial resolution, greatly enhancing image quality and intrinsic structural contrast and enabling the visualization and differentiation of diseased structures based on nuanced variations in signal intensity and contrast enhancement patterns, enabling pathologies such as tears to be seen with much greater clarity.^30^ MRI diagnosis is based on linear signal changes reaching the articular surfaces of the meniscus. Nevertheless, linear changes within the meniscus that are not in contact with the meniscal surfaces, such as grade I and II tears, which are not visible on arthroscopy, are unlikely to represent significant lesions compared with a meniscus without any interior changes on MRI.^31^ In our study, although not all cases included an exact classification grade (e.g., a specific grade such as grade IIb), the reports did indicate whether the tear reached the surface or was an incomplete intrasubstance lesion. This distinction allowed us to assess all surface tears, defined as grade III, in reference to arthroscopy, aligning with methods used in previous studies.^26^

The costs associated with MRI use in hospitals can be relatively high compared with other imaging modalities, such as computed tomography scans and plain radiographs.^32^ The high costs of using MRI scans stem from the fact that the equipment is more costly and that the process of undertaking MRI scans for patients requires and involves longer staff times.^32^ Furthermore, the scheduling wait times for MRI scans are longer than those for computed tomography and radiography scans, especially in large or government-funded public health systems, with wait times of up to 20 weeks.^33^ Thus, alternative cost-effective measures such as clinical examination testing alone with tests such as the McMurray, Thessaly, and joint-line tenderness tests may be warranted or more justified in health care contexts when access to MRI is limited or when cost constitutes a significant barrier.^34^ Clinical examination testing has been shown to be highly sensitive and specific in the assessment of meniscal tears, with the McMurray test having a reported sensitivity and specificity of 84% and 84.21%, respectively, for MM tears and 84.44% and 76.92%, respectively, for LM tears.^35^ Moreover, for medial joint-line tenderness, the sensitivity and specificity were 88.15% and 55.55%, respectively, for MM tears, whereas for lateral joint-line tenderness, the sensitivity and specificity were 86.95% and 66.66%, respectively.^35^ The Thessaly test was found to have the highest sensitivity and specificity of all the tests, with 92.10% sensitivity and 88.88% specificity for MM tears and 93.33% sensitivity and 84.16% specificity for LM tears with reference to arthroscopy.^35^ Therefore, there is a high utility for clinical examination testing in health care settings with obstacles to MRI access.

To build on our findings, future studies should focus on including standardizing reporting procedures and reducing the time interval between MRI and arthroscopy to enhance accuracy in assessing meniscal tear extent prior to arthroscopic confirmation. Conducting future research using a prospective design and implementing a more standardized data collection and reporting process will enhance consistency and comparability across analyses, thereby strengthening the reliability of results. By implementing these refinements, future studies can better validate and consolidate our conclusions, offering a more precise understanding of MRI’s diagnostic accuracy in detecting meniscal tears.

### Limitations

Several limitations may have impacted the findings of this study. In comparison with knee arthroscopy, the relatively lower diagnostic values of some parameters for MM and LM tears could be explained by radiology inter-operator variability in reporting MRI findings. Furthermore, different radiology operators’ and surgeons’ interpretations and techniques in detecting meniscal tears may have introduced inconsistencies in reporting findings. Moreover, the absence of a standardized procedure for reporting meniscal tear findings on MRI and arthroscopy, owing to the study’s retrospective nature, led to under-reporting of the grade, morphology, and location of meniscal tears. Finally, the prolonged interval of up to 6 months between MRI and arthroscopy may have allowed degenerative intrasubstance changes in the knee to progress into grade III tears that can be visualized by the time of arthroscopy, potentially impacting the accuracy of our findings.

## Conclusions

MRI is an effective first-line noninvasive diagnostic tool for investigating meniscal tears, with overall diagnostic accuracies of 88.64% for MM tears and 87.27% for LM tears. MM tears had the highest incidence, particularly in the posterior horn. MRI showed high sensitivity for MM tears, high specificity for LM tears, and substantial agreement with arthroscopy in diagnosing both MM and LM tears. However, MRI’s specificity for MM tears and sensitivity for LM tears were lower, suggesting that it may not be as reliable in confirming MM tears or ruling out LM tears.

## Disclosures

All authors (A.M.A.E-H., A.J.T., M.A.K., H.N.K., A.J.B.) declare that they have no known competing financial interests or personal relationships that could have appeared to influence the work reported in this paper.
